# Autochthonous Fermentation as a Means to Improve the Bioaccessibility and Antioxidant Activity of Proteins and Phenolic Compounds of Yellow Pea Flour

**DOI:** 10.3390/foods13050659

**Published:** 2024-02-22

**Authors:** María Agustina Cipollone, Analía G. Abraham, Ariel Fontana, Valeria A. Tironi

**Affiliations:** 1 Centro de Investigación y Desarrollo en Criotecnología de Alimentos (CIDCA), Centro Científico Tecnológico La Plata—Consejo Nacional de Investigaciones Científicas y Técnicas (CONICET), Comisión de Investigaciones Científicas de la Provincia de Buenos Aires (CICPBA), Universidad Nacional de La Plata (UNLP), 47 y 116, La Plata B1900AJJ, Argentina; maguscipollone@hotmail.com (M.A.C.); aga@biol.unlp.edu.ar (A.G.A.); 2 Área Bioquímica y Control de Alimentos, Facultad de Ciencias Exactas, Universidad Nacional de La Plata (UNLP), 47 y 115, La Plata B1900AJJ, Argentina; 3 Instituto de Biología Agrícola de Mendoza (IBAM), CONICET, Facultad de Ciencias Agrarias (FCA), Universidad Nacional de Cuyo (IBAM—CONICET—FCA—UNCuyo), Almirante Brown 500, Chacras de Coria M5528AHB, Argentina; afontana@mendoza-conicet.gob.ar

**Keywords:** yellow peas, natural fermentation, gastrointestinal digestion, antioxidant properties

## Abstract

This study focused on evaluating the potential of the natural fermentation of pea flour to improve the release of antioxidant compounds. Preliminary fermentations of 36.4% *w*/*w* flour dispersions were performed in tubes under different conditions (24 and 48 h, 30 and 37 °C). Finally, fermented flours (FFs) were obtained in a bioreactor under two conditions: 1: 36.4% *w*/*w*, 24 h, 30 °C (FF1); 2: 14.3% *w*/*w*, 24 h, 37 °C (FF2). The pH values decreased to 4.4–4.7, with a predominance of lactic acid bacteria. As in the fermentations in tubes, an increment in the proteolysis degree (TNBS method) (greater for FF2), polypeptide aggregation and a decrease in their solubility, an increase in <2 kDa peptides, and an increase in the Oxygen Radical Absorption Capacity (ORAC) potency of PBS-soluble fractions after fermentation were demonstrated. Also, fermentation increased the proteolysis degree after simulated gastrointestinal digestion (SGID, COST-INFOGEST) with respect to the non-fermented flour digests, with some differences in the molecular composition of the different digests. ORAC and Hydroxyl Radical Averting Capacity (HORAC) potencies increased in all cases. The digest of FF2 (FF2D) presented the greater ORAC value, with higher activities for >4 kDa, as well as for some fractions in the ranges 2–0.3 kDa and <0.10 kDa. Fermentation also increased the 60%-ethanol-extracted phenolic compounds, mainly flavonoids, and the ORAC activity. After SGID, the flavan-3-ols disappeared, but some phenolic acids increased with respect to the flour. Fermentation in condition 2 was considered the most appropriate to obtain a functional antioxidant ingredient.

## 1. Introduction

Legumes have high contents of protein, essential amino acids, fiber, B vitamins (including folic acid, thiamine, and niacin), and minerals. In comparison, legumes contain twice the amount of protein in whole-grain cereals (wheat, oats, and barley) and triple that in rice. In particular, peas (*Pisum sativum* L.) have a protein content of 20–23.5% *w*/*w* [1], which positions them as an important source of protein. Also, pulses are good sources of various phenolic compounds (PCs), including phenolic acids, flavonoids, isoflavones, and tannins. The contents and types of PCs, which can be free, esterified, or linked to other components, vary with the pulse type and genotype; for example, the PC composition changes with the seed coat color of the legume [2].

Some suitable options to increase the consumption of legumes in the daily diet are to use their flours in formulas for making baked goods and to improve their sensory and functional features through fermentation [3], which is one of the oldest biotechnological processes in the production of foods based on cereals and legumes. As indicated by Adebo et al. [4], fermented food products are sometimes classified as “functional foods” due to their potential health benefits. Natural or spontaneous fermentation is the most common means of sourdough preparation in developing countries. Sourdough consists of a mixture of flour and water fermented with the native microbiota of the seeds. This natural fermentation occurs through the sequential and competitive actions of an abundance of microorganisms, with the best-adapted strain(s) having a better growth rate, eventually dominating the microbiota [3,4]. The original microbiota of grains, and therefore flour, is affected by various factors, such as climate (temperature and humidity), storage conditions, insect attacks, and fungicide application. Another variable that affects the fermentation process is the type of grain due to differences in the quantity and quality of carbohydrates as fermentation starter substrates, in nitrogen sources, and in growth factors. This microbiota is composed of a great diversity of bacteria, especially lactic acid bacteria, (LABs), and yeasts [4]. Throughout the fermentation, the microorganisms most adapted to the environmental conditions will prevail, generally the LABs.

The technological parameters used in the preparation of fermented products, such as time, temperature, flour-to-water ratio (making fermentation occur in a solid or liquid medium), and agitation, also have a key influence on microbial communities. In this sense, fermented doughs or flours can be classified into three types: type I, prepared by a process of daily refreshments; type II, obtained in a single fermentation step, generally in a liquid medium in a bioreactor; and type III, corresponding to those of type II that are subsequently dried and stabilized. Each type will have different characteristics and uses, and the prevalence of different types of microorganisms has been shown [4,5,6].

The fermentation of seed-based products produces several shelf life, texture, flavor, and nutritional improvements. Several studies have demonstrated that fermentation (with starters or through the native flora) of legumes enhances their nutritive value, reduces some anti-nutritional endogenous compounds, such as phytic acid, exerts beneficial effects on protein digestibility and their biological value, and produces the release of bioactive compounds [4]. It has beneficial effects on irritable bowel syndrome since various compounds, including gluten, digestive enzyme inhibitors, and certain carbohydrates or fermentable polyols, can be hydrolyzed, preventing symptoms [4,7]. Moreover, it influences the bioavailability of phytochemicals, particularly phenolic compounds (PCs), by releasing bound or non-extractable PCs and their aglycones. Also, proteolysis occurs through endogenous seed proteases that can be activated by lowering the pH or by microbial proteases and peptidases. In addition to the functional, physical, and chemical aspects of protein modification, fermentation can produce the liberation of bioactive peptides by protein hydrolysis, which would confer positive effects on human health [8]. Regarding antioxidant activity, fermentation could produce an increase through different mechanisms, such as the release of antioxidant peptides and the release and/or transformation of PCs. It has also been reported that microorganisms could increase the antioxidant capacity of fermented products through the secretion of antioxidant enzymes, glutathione, and other biomolecules, such as exopolysaccharides, with this activity [4,8].

The literature on the antioxidant effect of pea-derived peptides is scarce. Therefore, a first evaluation of the antioxidant capacity of yellow pea flour and protein isolate was carried out before and after being subjected to a simulated gastrointestinal digestion process (SGID) [1]. Also, we have studied the profile and antioxidant activity of PCs and the effect of SGID (Cipollone et al., under revision). Interesting antioxidant activities of peptides and PCs after the gastrointestinal digestion of protein isolates and flour were demonstrated. Furthermore, while there is quite a bit of work on legume fermentation and some on pea fermentation with starters, so far, we have not found research specifically studying indigenous pea fermentation, let alone the effect on gastrointestinal digestion and antioxidant activity. In the present investigation, the natural fermentation of yellow pea flour was performed under different conditions, and its effects on the proteolysis, PC profile, and antioxidant activity of peptides and PC fractions were evaluated before and after SGID in order to obtain an ingredient with improved antioxidant activity potential.

## 2. Materials and Methods

### 2.1. Chemicals and Samples

Alpha-amylase from *Bacillus subtilis* (10,070, 57.4 U/mg), pepsin from porcine gastric mucosa P6887 (3200–4500 U/mg), porcine pancreatin 4XUSP P1750, bovine bile salts B3883, Trolox (6-hydroxy-2,5,7,8-tetramethylchroman-2-carboxylic acid), blue dextran, aprotinin, hippuric acid, 2,4,6-trinitrobencenesulfonic acid (TNBS), and AAPH (2,2′-Azo-bis-(2-methylpropionamidine) dihydrochloride) were purchased from Sigma Chemical Co., Ltd. (St. Louis, MO, USA). Fluorescein sodium and 2,2′-Azinobis-(3-ethylbenzthiazolin-6-sulfonic acid) (ABTS) were from Fluka (Steinheim, Germany). Nutritive agar (NA) was from Britania (Buenos Aires City, Argentine); Man, Rogosa, and Sharpe (MRS) medium and Chloramphenicol Yeast Glucose Agar (YGC) were from Biokar Diagnostics (Allone, France). Other reagents were of analytical grade.

Yellow pea seeds (*Pisum sativum* L. var. Yams) cultivated in Buenos Aires province (Argentine) in 2018 and 2019 were ground in a Udy mill (0.5 mm mesh) to prepare the flour.

### 2.2. Autochthonous Fermentation

#### 2.2.1. Preliminary Tests

A preliminary screening to analyze different fermentation conditions was carried out. In the first stage, the tests were carried out in tubes with agitation in a rotary shaker. Flour was dispersed in distilled water in a ratio of 1/1.75 (flour concentration: 36.4% *w*/*w*); two times (24 and 48 h) and two temperatures (30 and 37 °C) of incubation were evaluated. After fermentation, the samples were frozen. The final pH, proteolysis degree, total and soluble protein contents, and antioxidant activity (ORAC) of the four fermented flours were analyzed according to the methodologies described later.

#### 2.2.2. Preparation of Fermented Flour

After the preliminary tests, the fermentation process was performed in a bioreactor with a glass jacket connected to a water bath (LAUDA RMT6, Lauda-Königshofen, Germany) that allows the recirculation of thermostatic water. Agitation (250 rpm) was achieved by using a vertical stirrer (Dlab OS20-PRO20L; DLAB Scientific Co., Ltd., Beijing, China). Two tests were carried out under different conditions. In the first one, flour was dispersed in distilled water in a ratio of 1/1.75 (similar to tube assay), and the mixture was incubated at 30 °C for 24 h (FF1). In the second, the flour/distilled water ratio was 1/6, and the fermentation was conducted at 37 °C for 24 h (FF2). Dispersions of flour in distilled water without fermentation were used as controls (F1 and F2). pH measurements (Seven easy pH, Mettler-Toledo, Switzerland) were taken at different times during the incubations. After fermentation, the samples were lyophilized.

#### 2.2.3. Microbiological Analysis

The fermented (FF1/FF2) and non-fermented (F1/F2) samples were serially diluted in selective agar media for the isolation and counting of different microorganisms: MRS incubated for 48 h at 30 °C in anaerobiosis (AnaeroJar 2.5 L, Oxoid Ltd., Hampshire, UK); NA incubated for 24 h at 30 °C in aerobic conditions (F.A.C incubator, Pilar, Argentine); and YGC incubated for 48 h at 30 °C in aerobic conditions (F.A.C incubator). All the colonies obtained were tested by Gram staining and the catalase test.

### 2.3. Centesimal Composition

The protein content was determined by the micro-Kjeldahl method (f = 5.6 g protein/g N [9]) followed by colorimetric determination [10]; moisture and ash were determined according to AOAC 1984 (24.002 and 24.009) [11], and lipids according to AOAC 1990 (920.39) [12]. The Megazyme kit (Megazyme International Ltd., Wicklow, Ireland) was used to determine total dietary fiber (TDF) (AOAC 1995, 991.43) [13] using a VELP GDE enzymatic digester and a VELP CSF-6 dietary fiber extractor (VELP Scientifica, Usmate Velate (MB), Italy). Total carbohydrates were obtained by difference [14].

### 2.4. Simulated Gastrointestinal Digestion (SGID)

The protocol of Minekus et al. [15] was applied to fermented and non-fermented samples, obtaining the corresponding digests (FF1D, FF2D and F1D, F2D). The process was performed in a bioreactor with a glass jacket connected to a water bath (LAUDA RMT6) at 37 °C with agitation (90 rpm oral and gastric phases, 100 rpm intestinal phase; vertical stirrer, Dlab OS20-PRO20L, DLAB Scientific Co., Ltd., Beijing, China). The pH was monitored during the digestion process using a pH meter (Seven easy pH, Mettler-Toledo, Switzerland). Oral phase: Samples (about 50 g of FF1, FF2 and F1, F2) were homogenized with 35 mL of electrolyte solution for the simulated salivary fluid (SSF, 15.1 mmol/L KCl, 3.7 mmol/L KH_2_PO_4_, 13.6 mmol/L NaHCO_3_, 0.15 mmol/L MgCl_2_(H_2_O)_6_, 0.07 mmol/L NH_4_HCO_3,_ pH = 7), and 5 mL of α-amylase solution in SSF (26 mg/mL), 250 µL of 0.3 mol/L CaCl_2_, and 9.75 mL of H_2_O were added (all reactants were preincubated at 37 °C). The mixture was agitated and incubated for 2 min at 37 °C. Gastric phase: The oral solution was mixed with 75 mL of the electrolyte solution for the simulated gastric fluid (SGF, 6.9 mmol/L KCl, 0.9 mmol/L KH_2_PO_4_, 25 mmol/L NaHCO_3_, 47.2 mmol/L NaCl, 0.1 mmol/L MgCl_2_(H_2_O)_6_, 0.6 mmol/L NH_4_HCO_3_, pH = 3), 16 mL of pepsin solution (25,000 Anson U/mL in SGF), and 50 µL 0.3 mol/L CaCl_2_, adjusting the pH to 3 with 2 mol/L HCl and adding water to complete 100 mL of SGF. The mixture was incubated for 2 h at 37 °C. Intestinal phase: The gastric solution (200 mL) was mixed with 110 mL of the electrolyte solution for the simulated intestinal fluid (SIF, 6.8 mmol/L KCl, 0.8 mmol/L KH_2_PO_4_, 85 mmol/L NaHCO_3_, 38.4 mmol/L NaCl, 0.33 mmol/L MgCl_2_(H_2_O)_6_, pH = 7), 50 mL of the pancreatin solution (800 TAME U/mL in SIF), 25 mL of bovine bile salts (150 mg/mL), and 400 μL of 0.3 mol/L CaCl_2_; the pH was adjusted to 7 with 1 mol/L NaOH, and water was added to complete 200 mL of SIF. The mix was incubated for 2 h at 37 °C. After that, enzyme activities were inactivated by incubating the mixtures at 85 °C for 10 min. The electrolyte solutions for SSF, SGF, and SIF were prepared according to [15].

### 2.5. Characterization of Polypeptide/Peptide Fraction

(1) Protein hydrolysis degree (HD). The HD was measured by the 2,4,6-trinitrobencenesulfonic acid (TNBS) method [16,17]. The HD was calculated as follows (Equation (1)):

(1) $$\mathrm{HD}=\frac{\left(\left[-NH_{2}\right]h\right)}{\left([-NH_{2}]\propto \right)}\times 100$$

where [−NH_2_] indicates the concentration of free amino groups in the hydrolyzed samples (h). The parameter [−NH_2_]_∞_ was estimated according to Equation (2):

(2) $$\left[-NH_{2}\propto \right]=\frac{1}{\mathrm{Maa}}\times \left(1+\mathrm{flys}\right)\times \mathrm{Cprot}$$

where M_aa_ is the average molecular weight of amino acids (169.42 g/mol), flys is the proportion of lysine (1/17.8) (values calculated from amino acid composition of peas [18]), and C_prot_ is the protein concentration

(2) Glycine-SDS-PAGE [19]. Freeze-dried samples were dispersed in 0.0625 mol/L Tris-HCl, 2% SDS, and 10% *v/v* glycerol buffer (pH = 8.8) and centrifuged before loading in the gel. Separating and stacking gels (120 and 40 g/L acrylamide, respectively) were used. Runs were carried out in a Mini Protean II Dual Slab Cell (BIO-RAD, Hercules, CA, USA) device at room temperature, applying a constant current (30 mA/gel) in the case of SDS-PAGE and varying it between 30 mA/gel and 100 mA/gel after passing through the stacking gel for tricine-SDS-PAGE. Gels were stained with Coomassie Brilliant Blue R-250 (1 g/L). Silver staining was applied to increase the analytical sensitivity [20].

(3) Aqueous extraction. Suspensions (20 mg/mL) of freeze-dried samples in PBS (KH_2_PO_4_ 1.5 mmol/L, NaCl 138 mmol/L, KCl 3 mmol/L, Na_2_HPO_4_ 8.1 mmol/L, pH = 7.4) were prepared by agitation at 500 rpm (1 h, 37 °C) (Termomixer Eppendorf) and then centrifugation (10,000× *g*, 10 min, room temperature, Hermle Labortechnik GmbH, Wehingen, Germany). The soluble protein concentration was determined by the Lowry method [21].

(4) Gel filtration FPLC chromatography. Soluble fractions (see (3) in Section 2.5) were analyzed in an ÄKTA purifier (GE Healthcare, San Diego, CA, USA) device using a molecular exclusion column. Superdex Peptide 10/300 GL (GE Healthcare) (exclusion limit = 10 kDa; separation range = 0.1–7 kDa) was calibrated with blue dextran (exclusion volume Vo = 7.60 mL), aprotinin (6.5 kDa), vitamin B12 (1.35 kDa), and hippuric acid (0.18 kDa), obtaining the following calibration curve: log MM = 4.84–3.30 K_av_, where K_av_ = (V_e_ − V_o_)/(V_t_ − V_o_), V_e_ is the elution volume of the resolved species, V_o_ is the void volume, and Vt is the total volume of the column (V_t_ = 24 mL). Samples were filtered through a 0.45 µm nylon filter, and 200 µL of sample was loaded and eluted with PBS buffer at 0.5 mL/min for Superdex 30. Detection at 210 nm was performed. Fractions (500 µL) were collected.

### 2.6. Characterization of PCs

(1) Ethanol extraction. Extractions were performed according to previous optimization in our lab [unpublished]. Ultrasound-assisted extraction (UAE) in a VCX 750 ultrasonic processor (Sonics & Materials Inc., Newtown, CT, USA) was applied using a mixture of 60:40 ethanol/water and the following conditions: 15 min, 40% amplitude. The extractions were carried out in an ice bath to avoid excessive increases in temperature, which was maintained below 42 °C in all cases. The extracts were centrifuged (Hermle Labortechnik GmbH, Wehingen, Germany, 5 min, 10,000× *g*, room temperature). The supernatants were evaporated (30 °C, 2 h cycles, Concentrator plus/Vacufuge^®®^ plus, Eppendorf, Hamburg-Nord, Germany) and resuspended in PBS buffer.

(2) Total PC content (TPC). TPC was determined using the Folin–Ciocalteu method [22]. To 325 µL of sample, 50 µL of 1 eq/L Folin reagent was added and mixed by shaking, and 3 min later, 375 µL of 20% *w/v* Na_2_CO_3_ was added. The mixture was allowed to stand for one hour in the dark. After that, the absorbance at 760 nm was measured in a microplate reader (SYNERGY HT SIAFRT, Biotek Instruments, Winooski, Vermont, USA). In parallel, a standard curve was made with gallic acid (0–0.06 mg/mL). The results are expressed as mg gallic acid equivalent (GAE)/g sample in dry matter (dm). All determinations were performed at least in duplicate.

(3) Gel filtration FPLC chromatography. Ethanolic extracts were analyzed in accordance with what is described in item (4) of Section 2.5.

(4) Profiling and quantification of phenolic compounds.

For the determination of the qualitative and quantitative profiles of the PCs, dry extracts were dissolved in the initial mobile phase of the chromatographic method at the time of analysis. The separation and determination of PCs were performed in a high-performance liquid chromatograph coupled with diode-array and fluorescence (HPLC-DAD-FLD) detectors (Dionex Ultimate 3000 system, Dionex Softron GmbH, Thermo Fisher Scientific Inc., Germany) and a reversed-phase Kinetex C18 column (3.0 × 100 mm, 2.6 mm; Phenomenex, Torrance, CA, USA). The software Chromeleon 7.1 was used to control all the parameters of the system and to process the obtained data. The list of PCs determined and the chromatographic and detection conditions were those reported by Ferreyra et al. [23], with slight modifications. The mobile phases were aqueous solutions of 0.1% formic acid (eluent A) and acetonitrile (eluent B). The gradient applied was as follows: 0–1.7 min, 5% B; 1.7–10 min, 30% B; 10–13.5 min, 95% B; 13.5–15 min, 95% B; 15–16 min, 5% B; 16–19, 5% B. The flow rate was set at 0.8 mL/min, the column temperature was 35 °C, and the injection volume was 10 μL. The analytical flow cell for DAD was set to scan from 200 nm to 400 nm; a data collection rate of 5 Hz, a bandwidth of 4 nm, and a response time of 1 s were used. Different wavelengths (254, 280, 320, and 370 nm) were used according to the maximum absorbance of analytes for DAD. For FLD, an excitation wavelength of 290 nm and a monitored emission response of 315, 360, or 400 nm were used, depending on the targeted analytes. A data collection rate of 10 Hz was used for FLD. The retention times of compounds in the samples were compared with those of authentic standards for the identification of PCs. Calibration plots for the studied analytes showed linear ranges between 0.05 and 40 mg/L (r^2^ > 0.993) for most of the analytes.

### 2.7. Antioxidant Activity

(1) Oxygen Radical Absorbance Capacity (ORAC). The ORAC assay was carried out using previously optimized protocols in our laboratory [24]. A 53.3 nmol/L fluorescein solution in phosphate buffer (150 μL) was mixed with 25 μL of sample or the same volume of either the phosphate buffer (negative control) or Trolox (positive control) and then preincubated at 37 °C for 10 min; 160 mmol/L AAPH (25 μL) in phosphate buffer was added, and the reaction mixture was incubated at 37 °C for 45 min. The fluorescence intensity (λ_exc_: 485; λ_em_: 535 nm) was read every min in a SYNERGY HT–SIAFRT™ multidetection microplate reader (Biotek Instruments, Winooski, VT, USA) to obtain the fluorescein decay curve. The area under the curve was obtained according to Equation (3):

AUC = 0.5 + f1/f0 + f2/f0 + … + fi − 1/f0 + 0.5 fi/f0(3)

where f is the fluorescence value at a particular time during the decay. A blank without AAPH was included, and % scavenging was calculated as follows:

% ROO · scavenging = [(AUC_S_ − AUC_CN_)/(AUC_B_ − AUC_CN_)] × 100 (4)

where S = sample, B = blank, and NC = negative control. Trolox (6.25–75.0 μmol/L) was used as a reference compound. The concentration that inhibits 50% of radicals (IC_50_) was obtained from dose–response curves. PBS and separated FPLC fractions as well as ethanolic extracts were analyzed.

(2) Hydroxyl Radical Averting Capacity (HORAC) [24]. The hydroxyl radical was generated by a cobalt-mediated Fenton-like reaction, with fluorescein used as a probe. Either samples or buffer (20 μL) was mixed with 190 μL of 60.3 nmol/L fluorescein solution in phosphate buffer, and 15 μL of a 0.75 mol/L H_2_O_2_ solution and 75 μL of the cobalt solution (10 mg of picolinic acid and 11 mg of CoCl_2_.6H_2_O in 50 mL of water) were added. The mixture was incubated at 37 °C for 3 h in the SYNERGY HT microplate reader; the fluorescence (λ_exc_: 485; λ_em_: 535 nm) was read at 1 min intervals to obtain the AUC (Equation (3)). The % OH· inhibition was calculated according to Equation (4). Chlorogenic acid (0.05–0.5 mg/mL) was used as a reference compound. The concentration that inhibits 50% of radicals (IC_50_) was obtained. PBS fractions were analyzed.

(3) ABTS free radical scavenging capacity. The ABTS^•+^ radical cation decolorization method was carried out according to Tironi and Añón [25], with some modifications. The ABTS^•+^ radical was obtained by reacting a 7 mmol/L solution of ABTS with potassium persulfate (final concentration: 2.45 mmol/L) and incubated at room temperature in the dark for at least 16 h. Prior to the assay, the ABTS^•+^ solution was diluted in PBS to obtain an absorbance at 734 nm of 0.70 ± 0.02 in a 1 cm cuvette with a Beckman DU 650 spectrophotometer (Beckman Coulter, Brea, CA, USA). Mixtures of 1000 µL of the diluted ABTS^•+^ solution with 100 µL of the resuspended extracts were prepared in 48-well plates, and the absorbance at 734 nm was measured at various times (0–15 min) in a SYNERGY HT SIAFRT microplate reader (Biotek Instruments). Trolox (0.05–0.20 mmol/L) was used as the reference compound. With the measurements made for each sample and the blank (PBS buffer), the % inhibition of the radical was calculated using Equation (5):

% ABTS^•+^ scavenging = [(Abs_B0_ − Abs_S15_) − (Abs_B0_ − Abs_B15_) × 100]/Abs_B0_ (5)

where Abs_B0_ and Abs_B15_ refer to the absorbance of the blank at 0 and 15 min, respectively, and Abs_S15_ refers to the absorbance of the sample at 15 min. Ethanolic fractions were analyzed.

### 2.8. Statistical Analysis

Determinations were performed on at least two independent batches. Differences between samples were analyzed by one-way ANOVA multiple comparisons. Significant differences (*p* < 0.05) among mean values were evaluated by the Tukey HSD test (Statgraphics Centurion XVI).

## 3. Results and Discussion

### 3.1. Preliminary Assays on Natural Fermentation of Yellow Pea Flour

Our first studies on the natural fermentation of yellow pea flour were related to the evaluation of fermentation conditions that produced a certain degree of proteolysis with the potential release of antioxidant peptides. In this way, dispersions containing 36.4% *w*/*w* flour and four time/temperature pairs were evaluated (24 and 48 h, 30 and 37 °C). The final pH, proteolysis degree (HD), protein solubility, and antioxidant activity (ORAC, PBS-soluble fractions) of the four fermented flours were analyzed (Table 1). The final pH value achieved was different according to the incubation time, as was expected, reaching 3.6 and 3.9 ± 0.1 after 48 h at 30 and 37 °C, respectively, and 5.1 ± 0.1 and 4.7 ± 0.2 after 24 h at 30 and 37 °C, respectively, showing no significant differences (*p* > 0.05) between the two incubation temperatures at any time. In addition, no significant (*p* > 0.05) differences in the proteolysis degree (HD) and significant (*p* < 0.05) but small differences in the ORAC IC_50_ value were obtained among the different fermented products (Table 1). According to these results, it was decided to continue working with fermentations conducted for 24 h (30 and 37 °C) since the prolongation of the time to 48 h did not generate significant changes either in HD % or in the solubility of polypeptides/peptides and only very minor differences in ORAC activity.

### 3.2. Preparation of Fermented Flours in Bioreactor

Taking into account the information obtained in the preliminary tests, natural fermentation was carried out in a bioreactor. Two different systems were studied:

Test 1. The fermented flour FF1 was obtained by using a 36.4% *w*/*w* flour dispersion and incubating it for 24 h at 30 °C. The evolution of the pH was recorded, and the value dropped from 6.2–6.3 (t = 0) to a final value (24 h) of 4.75 (Table 2). Thus, pH values decreased to a greater extent than in tube fermentation in the same conditions (Table 1). It should be noted that this dispersion presented a high viscosity and, in consequence, was difficult to stir.

Test 2. The fermented flour FF2 was obtained by using a lower flour concentration (14.3% *w*/*w*) and incubating it for 24 h at 37 °C to achieve a greater fluidity of the dispersion and easy agitation. The pH value achieved after 20 h of incubation was 4.75 (similar to that reached at 24 h by FF1), and after 24 h, it was about 4.4 (Table 2), showing a slightly higher decline (*p* < 0.05) than in FF1. These results suggest an increase in the fermentation rate under conditions of a lower flour/water ratio. Sáez et al. [26] reported a pH decrease from 6.30–6.43 to 4.80–4.83 after the first back-slopping for the natural fermentation (24 h at 37 °C) of different varieties of beans in 1 g/mL flour/water dispersions.

A microbiological screening of the non-fermented and fermented samples using nutrient agar (NA) (total mesophilic aerobic bacteria), YGC (fungi and yeasts), and MRS with a selection factor to observe the growth of LABs was performed. The microbiological counts of samples before fermentation (t = 0) were in a range of 4.2 to 4.5 log CFU/g in both NA and MRS, with no evident growth on the YGC medium (Table 2). These results are comparable to the previously reported value of 4.6 log CFU/g for unfermented chickpea flour [27] and are slightly higher than those obtained by Rizzello et al. [28] for Faba bean flour (3.6 log CFU/g). After fermentation, counts of around 9 log CFU/g (NA and MRS) were registered for FF1 and FF2 (Table 2). The bacterial colonies that grew in the MRS medium presented smooth edges, a white color, and a creamy appearance. When observed under the microscope, these colonies presented chained coccus-type and bacilli morphologies. According to *Bergey’s Manual*, Gram staining (positive) and the catalase test (negative) carried out on different colony-forming units indicated that these colonies could be presumptively identified as LABs.

Generally, sourdough contains a variable number of LABs, ranging from 7 to 9 log CFU/g [29]. Values of around 8 log CFU/g have been reported for different beans’ sourdoughs after 6 days of fermentation [26]. No growth was observed in the YGC medium, indicating undetectable counts of yeasts after the fermentation process in the lower dilution performed. At this point, it is worth mentioning that tests 1 and 2 were carried out at different times, which forced us to use different batches of peas (different harvest years and different storage times). However, the microbiological counts were similar, both for the initial microbiota and for the fermented samples. In this way, according to our experience and bibliographic data, the counts of microorganisms (LABs, yeasts) after fermentation do not present major differences between different harvests. However, it is very likely that there is a difference between the type and proportion of constituent microbial species, an aspect that will be studied in greater depth soon.

### 3.3. Composition of Fermented Flours

The macro-component compositions of the freeze-dried fermented flours were determined and compared with those of the non-fermented flours (Table 3). There were no significant differences (*p* > 0.05) in the ash and lipid contents for any of the samples, with values comparable to those previously reported for Canadian peas [18]. Fermented flours did not present significant changes in the protein content with respect to the corresponding non-fermented samples. Regarding the dietary fiber, the content obtained for F1 and F2 was comparable to others previously reported for pea seeds (15.3%, [30]). In relation to the effect of fermentation on this component, there was a significant increase (*p* < 0.05) in the case of FF2. It has been reported that the spontaneous fermentation of mung bean increased the crude fiber content [31]. However, another study [32] reported that pea, chickpea, and grass pea flours containing high levels of dietary fiber did not show significant variations after the fermentation process (*Lactiplantibacillus plantarum* or *Levilactobacillus brevis*, 24 h, 30 °C). Also, the natural fermentation of lupin and soy did not affect the contents of soluble, insoluble, and total fiber [33]. Further studies—which are not the subject of this work—will be necessary to analyze the effect of fermentation on the fiber composition and try to explain the small increase recorded for FF2 and its potential health benefit.

### 3.4. Changes in the Protein Fractions of Fermented Flours

As in the case of 10 mL (tube) fermentations, partial proteolysis was evidenced by the increment in the HD value. Although FF1 and FF2 presented similar HD values, we can remark that the HD value doubled in the case of FF1 and increased by 5 times in the case of FF2 with respect to the corresponding initial values (Table 4). In this way, there was a greater level of protein hydrolysis in the case of FF2.

A complementary test was carried out to determine whether endogenous proteases from pea seeds could be activated by the drop in pH, producing proteolysis. The mobilization of storage proteins in germinating seeds is initiated by endo-proteases that convert water-insoluble storage proteins into soluble peptides. Most of the plant proteases are neutral or alkaline, and there are few acid proteases (pH optimum: 2–3) widely distributed in the plant seeds [34]. In the case of cereals, the comparison of wheat and rye sourdoughs and chemically acidified doughs indicated that primary proteolysis is mainly attributable to endogenous proteases [35]. To evaluate this, the pH of a flour dispersion (14.3% *w*/*w*) was lowered with 2 mol/L HCl to the final value obtained in the fermentations (4.4), and the degree of protein hydrolysis was measured (TNBS method). A very low value (close to 0), even lower than those registered for flour dispersions in water before fermentation, was obtained. According to this, no activation of endogenous proteases was evidenced. In agreement, Akhtaruzzaman et al. [36] extracted proteases from seven overnight-imbibed leguminous seeds and found that the alkaline proteases involved in all seeds were more potent than the acidic proteases. In consequence, proteolysis in the fermented samples would be the product of the action of proteases from microorganisms. LAB strains displayed a wide range of proteolytic activities [27].

Comparing the results obtained for 10 mL (tube) fermentations and those in the reactor under the same conditions (36.4% *w*/*w*, 24 h, and 30 °C), there was no significant difference (*p* > 0.05) in the protein solubility values (52 and 56%, respectively, Table 1 and Table 4). In all cases, the solubility decreased in the fermented samples with respect to the non-fermented ones, which could be due to the formation of aggregates during the fermentation process, as will be discussed next.

**Figure 1:**
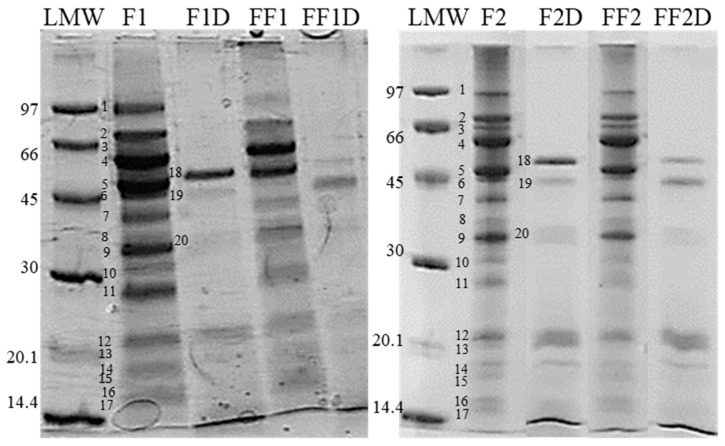
Electrophoresis SDS-PAGE of freeze-dried samples solubilized in electrophoresis buffer. F1: 36.4% *w/v* flour dispersion; F2: 14.3% *w/v* flour dispersion; FF1: fermented flour in condition 1 (36.4% *w/v* flour dispersion, 24 h, 30 °C); FF2: fermented flour in condition 2 (36.4% *w/v* flour dispersion, 24 h, 37 °C); F1D: F1 after SGDI; F2D: F2 after SGDI; FF1D: FF1 after SGDI; FF2D: FF2 after SGDI. LMW: low-molecular-weight standard (kDa).

The changes in the peptide/polypeptide profile produced by fermentation were analyzed by glycine-SDS-PAGE. The glycine-SDS-PAGE profiles of F1 and F2 (Figure 1) showed a great variety of polypeptides between 14 and 97 kDa. It was possible to detect bands tentatively belonging to linoleate 9S-lipoxygenase (band 1, 93 kDa), alpha-dioxygenase (band 2, 77 kDa), convicilin (an important storage protein in peas, 70 kDa, band 3), legumin subunits (59 kDa, band 4), free acidic (40 kDa, band 7) and basic (band 13, 20 kDa) legumin subunits, and vicilin subunits (53 kDa, band 5; 34 kDa, band 9: pea vicilin is heterogeneous, so variable polypeptides could be produced by different gene coding), and band 6 (probably alpha-galactosidase, 45 kDa); bands 11 and 12 (28 and 25, probably subunits/polypeptides of albumin-2), and bands 14 to 17 (20–14 kDa) would correspond to albumins. The recognition of the pea polypeptides was carried out according to Ma et al. [37].

After fermentation, a decrease in the intensity of all bands was observed, being more evident for 93 kDa (band 1) and for bands corresponding to MW < 40 kDa. Also, an increase in high-MW molecules that did not enter the gel could be observed in fermented samples, suggesting the presence of aggregates that remain even in the presence of SDS and urea (Figure 1). The formation of aggregates could explain the decrease in solubility observed in fermented samples (Table 4); this fact can be at least partially explained since pea proteins have their minimum solubility at the isoelectric point (between 4 and 5), coinciding with the final pH value in fermented flours. Band 10 (31 kDa, which could correspond to the anti-nutritional factor lectin [37]) appeared much more intense in samples F2 and FF2 than in F1 and FF1, while band 12 (25 kDa, which could include Kunitz-type trypsin inhibitor-like 2 protein [37]) has a higher intensity for F1 and FF1 with respect to F2 and FF2 (Figure 1). Beyond these differences between the two dispersions, the intensity of these bands decreased after fermentations, suggesting a diminution in the mentioned anti-nutritional factors. The reduction in the color intensity of several bands after fermentation could be associated with polypeptide diminution due to proteolytic activity. Byanju et al. [38] also observed this pattern of band discoloration in pea, lentil, and soybean flours after fermentation with *L. plantarum* and *Pediococcus acidilactici*. Also, the fermentation of pea flour with three LABs (*Pediococcus pentosaceus*, *Lactococcus raffinolactis*, and *L. plantarum*) resulted in similar patterns of the Coomassie brilliant blue-stained gels, which were not very different from the extract of the unfermented flour, except for the disappearance of some high-molecular-weight bands [39].

**Figure 2:**
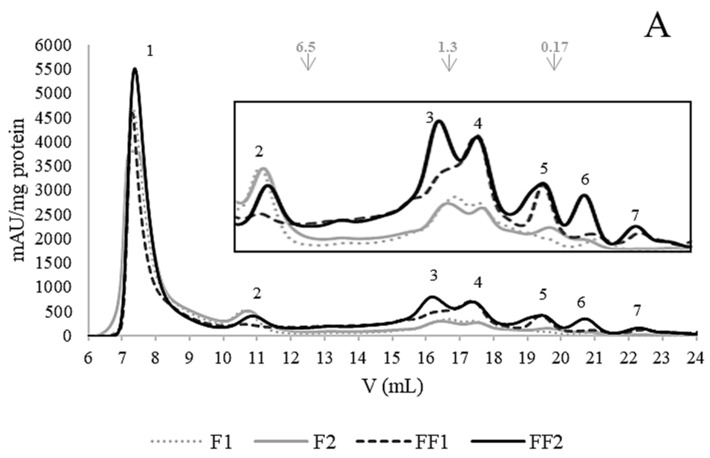

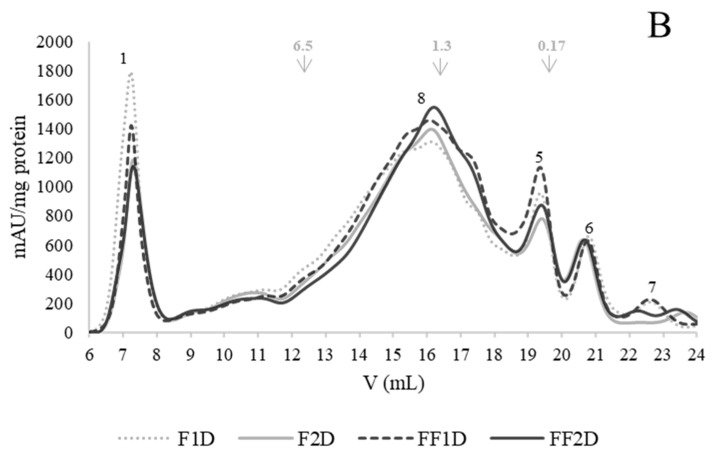
Gel filtration (FPLC) chromatograms (Superdex 30 column, optimal separation range < 10 kDa) of PBS-soluble fractions. (**A**) F1: 36.4% *w/v* flour dispersion; F2: 14.3% *w/v* flour dispersion; FF1: fermented flour in condition 1 (36.4% *w/v* flour dispersion, 24 h, 30 °C); FF2: fermented flour in condition 2 (36.4% *w/v* flour dispersion, 24 h, 37 °C). (**B**) F1D: F1 after SGDI; F2D: F2 after SGDI; FF1D: FF1 after SGDI; FF2D: FF2 after SGDI. Molecular weight markers (kDa) are shown at the top of chromatograms.

The peptide/polypeptide composition of the soluble fractions of non-fermented and fermented flours was analyzed by gel filtration chromatography using a Superdex 30 column (optimal separation in the range for MW < 10 kDa) in order to evaluate low-MW peptides. As expected, the chromatograms of F1 and F2 (Figure 2A) were similar. Fermentation caused an increase in molecules smaller than 6.5 kDa in both conditions (FF1 and FF2). However, some differences between FF1 and FF2 could be described: peak 2 (MW > 6.5 kDa) decreased more in the case of FF1, while peaks 1 (MW > 10 kDa), 3 (1.5–0.8 kDa), 5 (0.47–0.18 kDa), and 6 (0.18–0.08 kDa) increased more in the case of FF2 (with respect to the non-fermented flour), showing a greater occurrence of small molecules in FF2.

According to the electrophoresis and FPLC analyses, fermentation produced some minor changes in the protein profile of the pea flour related to the appearance of aggregates and soluble proteolytic fragments with MW < 2 kDa, with some differences between the two fermentation conditions assayed. These results, together with the registered proteolysis degree, showed that the LAB strains present in the fermented flours produced moderate proteolysis of the pea proteins.

### 3.5. Effect of Fermentation on Protein Fraction Bioaccessibility (SGID) and Antioxidant Activity

After SGID, as expected, HD significantly increased (*p* < 0.05) for all samples (Table 4). However, the values were significantly greater (*p* < 0.05) when the flour was previously fermented (FF1D and FF2D with respect to F1D and F2D, respectively), indicating that the fermentation process improved the proteins’ gastrointestinal digestion. FF1D presented a significantly greater (*p* < 0.05) HD value than FF2D. However, the HD value of FF1D was 7.5 times greater than that of F1, while the HD value of FF2D was 13 times greater than that of F2, showing a greater proportion of proteolysis in the second case (Table 4). SDS-PAGE (Figure 1) showed that in the samples subjected to SGID, most of the polypeptides disappeared, with the appearance of some bands, such as 18 (51 kDa), 19 (43 kDa), and 20 (a broad band of about 35 kDa), and partially remaining bands of MW < 25 kDa (legumin subunits and albumins) for all the digests (F1D, F2D, FF1D, and FF2D). In this way, some pea polypeptides resisted gastrointestinal digestion. This fact has been previously observed when the gastrointestinal digests of flours and protein isolates from two pea varieties were analyzed [1]. Ma et al. [37] reported that a pea protein hydrolysate obtained by the action of a mixture of trypsin, chymotrypsin, and peptidase presented a reduction in most of the bands present in the raw pea profile but with the persistence of bands with MWs between 10 and 30 kDa. Some differences could be detected among digests, mainly in the molecules generated by SGID. The intensity of bands 18 and 20 was greater for digests from non-fermented flour (F1D and F2D), while the intensity of band 19 was greater for digests from fermented flour (FF1D and FF2D) (Figure 1). In analyzing the effect of fermentation on the subsequent SGID, the electrophoretic profiles showed a lower intensity in some of the remaining bands in the fermented meals, in agreement with the highest HD values obtained for digests of fermented flours. Partial proteolysis due to the fermentation process made the sequences more susceptible to further degradation by the digestive enzymes, as has been previously reported [40].

In analyzing the composition of the PBS-soluble fractions of gastrointestinal digests, gel filtration chromatograms (Figure 2B) showed that the peaks corresponding to the exclusion volume (>10 kDa) decreased with respect to the undigested samples and significantly increased the number of molecules smaller than 6.5 kDa in all digested samples. Similar behavior has been previously reported for flours and protein isolates of two pea varieties and their corresponding digested samples [1]. Considering each particular peak, only minor differences in the area were observed among the four digests. Peak 8 (0.4 to 8 kDa) constituted the greatest modification after gastrointestinal digestion and presented the highest area in the four digests, representing about 60–63% of the total area. Peak 1 (the remaining MW > 10 kDa molecules) accounted for around 30–33% of the area, with F1D presenting the highest value and FF1D the lowest one. Peak 5 (0.47–0.18 kDa) represented about 2.5 to 4%, and peak 6 (< 0.18 kDa) between 2.5 and 3%.

The antioxidant activity of the PBS-soluble fractions of non-fermented and fermented pea flour before and after SGID was evaluated. The ORAC assay method measures the scavenging capacity against peroxyl radicals (generated from AAPH at 37 °C) by the oxidative degradation of fluorescein [40]. Dose–response curves for ORAC (ROO· scavenging % versus peptide concentration) were obtained, and IC_50_ values were calculated (Table 4). The ORAC activity was significantly (*p* < 0.05) increased by the fermentation process, with a diminution in IC_50_ values of 2.5 times for FF1 with respect to F1 and 2.7 times for FF2 with respect to F2, with a significantly (*p* < 0.05) lower IC_50_ value for FF2 (Table 4). Also, the HORAC assay was performed, in which the oxidative degradation of fluorescein is caused by hydroxyl radicals generated by the Fenton reaction [41]. Dose–response curves presented a linear fitting in this case. There was no significant difference (*p* > 0.05) between the IC_50_ values of non-fermented and fermented flours in any fermentation condition (Table 4), indicating that fermentation had no effect on this activity.

SGID produced a significant increase (*p* < 0.05) in ORAC activity in the case of both F1D and FF1D, with increases of about 4 and 9 times with respect to F1, respectively. F2D and FF2D also presented a significant increase (*p* < 0.05) in ORAC activity with respect to the initial sample (F2), with a potency increment of 5 times. FF2D presented an IC_50_ value that was slightly (but significantly) lower than that of FF1D (Table 4). The SGID process produced an increase in antioxidant HORAC potency since the IC_50_ values were reduced by half, without a significant difference between the different digests (*p* > 0.05). Based on these results, we can conclude that the natural fermentation of pea flour produced an increase in ORAC activity associated, in principle, with the release of peptides, but had no noticeable effect on HORAC activity. The difference in the sensitivity and in the mechanisms of action related to these two methods could explain the differences in the behavior of fermented flours.

**Figure 3:**
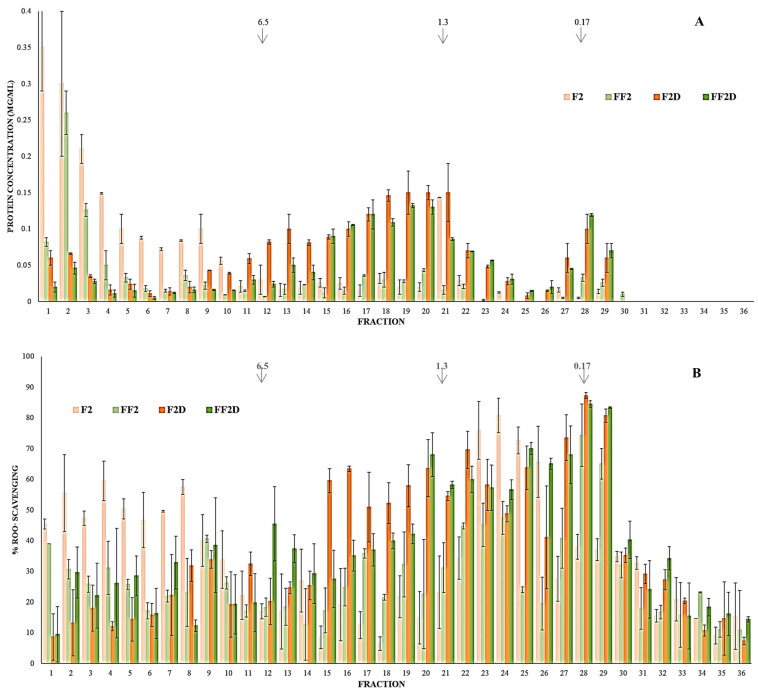
FPLC fractions separated from F1 (36.4% *w/v* flour dispersion), F2 (14.3% *w/v* flour dispersion), FF1 fermented flour in condition 1 (36.4% *w/v* flour dispersion, 24 h, 30 °C), and FF2 fermented flour in condition 2 (36.4% *w/v* flour dispersion, 24 h, 37 °C); F1D: F1 after SGDI; F2D: F2 after SGDI; FF1D: FF1 after SGDI; FF2D: FF2 after SGDI. (**A**): Protein concentration (Lowry method). (**B**): % ROO˙ scavenging (ORAC method).

Taking into account the previous results and some practical considerations related to the ease of agitation and dispersion, it was decided to continue studying the flour fermented in condition 2 (14.3% *w*/*w*, 24 h, and 37 °C). In order to learn more about the distribution of molecules that contribute to the antioxidant activity of these samples, fractions of different MWs from F2, FF2, F2D, and FF2D were separated by FPLC gel filtration, after which their peptide concentrations and ROO· scavenging activities were determined using the ORAC test (Figure 3). In F2, as expected, the fractions with the greatest polypeptide concentrations were those with MW > 10 kDa (fractions 1 to 9). These fractions presented ROO· scavenging activity (40–60%); however, fractions 23–26 (MWs between 0.29 and 0.59 kDa) presented the highest activities (66 to 81%, Figure 3A), but low or non-detectable concentrations of peptides (Figure 3B). According to the MWs of these fractions, they could involve peptides of between 3 and 5 amino acids, although the presence of other components, such as phenolic compounds, cannot be ruled out, all of which would present significant ORAC activity.

After fermentation (FF2), fractions 1 to 8 (>10 kDa) decreased their polypeptide concentrations (Figure 3A) and their ORAC activity (Figure 3B). Also, fractions 23 to 26 (0.29–0.59 kDa) had diminished ROO· scavenging activity, while several fractions in the ranges of 0.75 to 4 kDa (fractions 15–22) and 0.18–0.23 kDa (fractions 28–29, 65–74%) increased it (Figure 3B). In this way, the increment in ORAC activity registered after the fermentation of pea flour could be mainly related to the appearance of molecules in the ranges of 0.75–4 kDa and 0.18–0.3 kDa with improved ROO· scavenging. Most of the studies involving the formation of bioactive peptides by fermentation were carried out with LABs, which possess a complex system of proteases and peptidases [42]. As reported by Venegas-Ortega et al. [43], the differences found in LAB proteinases explain the variety of bioactive peptides produced, even when the same protein matrix is used. In a previous work [44], nine *Lactobacillus* strains were evaluated for their ability to grow in a pea seed protein-based medium and to hydrolyze purified pea proteins to produce peptides with antioxidant activity. Two strains, *Lacticaseibacillus rhamnosus* BGT10 and *Lacticaseibacillus zeae* LMG17315, exhibited strong proteolytic activity against pea proteins. These authors showed that the antioxidant activity (DPPH assay) of the fraction with MW < 10 kDa increased after 12 h of fermentation with *L. rhamnosus BGT10*. This fraction presented antioxidant activity in different assays, and when performing a separation by ion exchange chromatography, they showed that a low-abundance sub-fraction of basic peptides presented the highest activity.

The SGID process (F2D and FF2D) produced increases in the peptide concentrations of all fractions with MW < 3 kDa (Figure 3A) and increments in the ROO· scavenging % for almost all fractions with MW < 6.5 kDa (Figure 3B). F2D showed the higher scavenging % values (41–87%) for fractions between 0.14 and 4 kDa (fractions 15–29). FF2D presented higher scavenging values with respect to F2D in almost all fractions greater than 4 kDa (<45% scavenging) and in some of the fractions in the ranges of 2 to 0.3 kDa (18–26) and less than 0.10 kDa (<40% scavenging), and both digests presented their maximum ROO· inhibition in fractions of around 0.14–0.18 kDa (28 and 29, probably free amino acids), being 84 and 87% for FF2D and F2D, respectively (Figure 3B). These results also showed some differences in the molecular composition of the gastrointestinal digest of non-fermented and fermented pea flours.

### 3.6. Effect of Fermentation on PC Bioaccessibility (SGID) and Antioxidant Activity

Given the importance that PCs have in antioxidant activity, whether fermentation modified the content of PCs was evaluated on 60% ethanol extracts of F2 and FF2. The fermentation process significantly (*p* < 0.05) increased (about 3 times) the TPC measured by the Folin–Ciocalteu method (Table 5).

Gan et al. [45] reported that natural fermentation increased the TPC in most legumes, especially in the mottled cowpea, where it increased by about 80%. Xiao et al. [46] performed extractions with different solvents (80% methanol, 80% ethanol, 80% acetone, and water) of fermented mung bean, and in all of them, the TPC increased with respect to the non-fermented samples. These authors suggested that the chemical structures, polarities, and solubilities of the mung bean PCs were significantly influenced by the fermentation process. The PC profile of FF2 was analyzed by HPLC-DAD-FLD and compared to that of F2 (Table 6).

The PC composition of yellow pea flour has been previously studied in our lab (Cipollone et al., under revision), with (−)-epigallocatechin (a flavan-3-ol) and polydatin (stilbene) as the major PCs. Several changes were observed after fermentation. Increments in ellagic, rosmarinic, and especially caffeic acids but diminutions in gallic (a hydroxybenzoic acid), *p*-coumaric, and ferulic acids (hydroxycinnamic acids) were observed. OH-tyrosol was not detectable after fermentation. Among the flavonoids (the majority in the flour), only (−)-epicatechin and naringenin (flavanone) decreased, while the flavanone hesperitin, the flavan-3-ols (−)-epigallocatechin and (+)-catechin, and the flavonols rutin (quercetin-3-O-rutinoside), quercetin-3-glucoside, kaempferol-3-glucoside, and quercetin-3-glucoside increased; quercetin, which was not found in the flour, appeared in FF2 (Table 6). The total amounts of HPLC-DAD-FLD-detected PCs increased after fermentation, mainly due to a flavonoid family increment. Dueñas et al. [47] carried out fermentations of cowpea flour, both spontaneous and with *L. plantarum* ATCC 14,917 (48 h, 37 °C); both fermentations modified the contents of PCs but in a different manner. They found—as in our case—that the fermentation gave rise to the appearance of some PC compounds not detected in raw flour, such as quercetin. That was explained by the fact that pH lowering could activate some enzymes that hydrolyze quercetin glycosides, thus yielding quercetin. Lactobacillaceae possess a broad spectrum of enzymatic activities for the biotransformation of dietary PCs that could have participated in the change in the PC profile previously described. Esterases, reductases, and decarboxylases would participate in the conversion of hydroxycinnamic and hydroxybenzoic acids. In addition, LABs contain glycosyl hydrolases that seem to be dedicated to the hydrolysis of glycosides of plant secondary metabolites, such as glycosylated flavonoids, although little is known about the substrate specificity of these enzymes [48].

**Figure 4:**
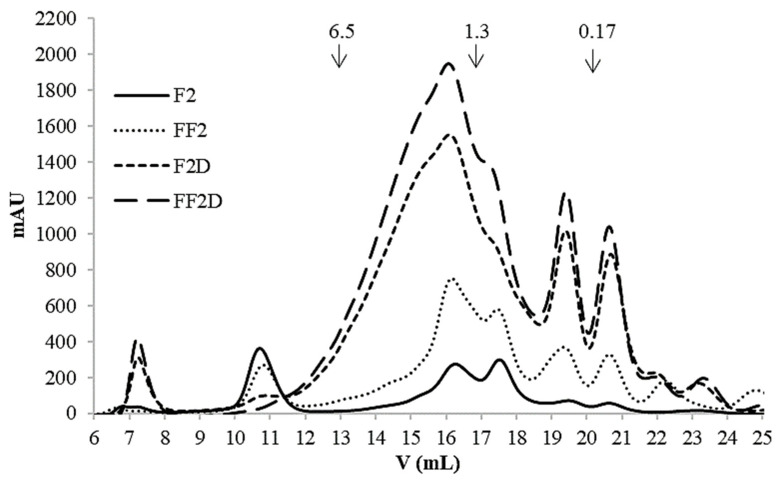
Gel filtration (FPLC) chromatograms (Superdex 30 column, optimal separation range < 10 kDa) of 60% ethanol extracts from F2: 14.3% *w/v* flour dispersion; FF2: fermented flour in condition 2 (36.4% *w/v* flour dispersion, 24 h, 37 °C); F2D: F2 after SGDI; FF2D: FF2 after SGDI. Molecular weight markers are shown at the top of chromatograms.

When analyzing the antioxidant activity of the ethanolic extracts, it was observed that the ABTS activity significantly decreased (*p* < 0.05) after fermentation (Table 5). In addition, fermentation did not have an effect on ROO· scavenging since FF2 presented a similar (*p* > 0.05) IC_50_ value for ORAC to that of F2 (Table 5). This behavior was different from that recorded for the fractions soluble in PBS, in which ORAC activity increased after fermentation (Table 4). Gel filtration FPLC chromatograms (Figure 4) of the ethanol extracts showed that both F2 and FF2 presented molecules with MWs in a broad range (<10 kDa), but they did not contain the larger polypeptides (>10 kDa) that appeared in the peak corresponding to the exclusion volume (unlike the fractions soluble in PBS, Figure 2). As in the PBS-soluble fractions, the increment in molecules lower than 2 kDa was evident after fermentation (Figure 4).

After SGID, an increase in the TPC was observed in F2D and FF2D with respect to their non-digested samples, being greater when the flour had been previously fermented (Table 5). According to this, Ketnawa and Ogawa [49] reported an increase in TPC values after subjecting fermented soybeans to an SGID process. SGID produced several changes in the PC profile of F (Cipollone et al., under revision). The gastrointestinal digest of fermented flour (FF2D) presented higher contents of some phenolic acids than F2D, such as gallic, syringic, caffeic, and rosmarinic acids (Table 6). However, the greatest difference was found in flavonoids, whose content was much lower in FF2D since compounds from the flavan-3-ol family (catechins and procyanidin) were not found. These results suggest that, after fermentation, these compounds were more available for the modifications that can occur during the gastrointestinal digestion process, such as the instability of catechins at neutral pH [50] and of procyanidin at gastric acid pH [51]. SGID produced significant decreases (*p* < 0.05) in the IC_50_ values of both digests, without significant differences between them (Table 5). It also led to an increase in ABTS activity in the case of FF2, with both digests showing similar IC_50_ values. Thus, although fermentation produced modifications in the PC profile of F, these did not translate into important changes in ORAC and ABTS activities after SGID. Sancho et al. [52] measured the antioxidant activity in the methanol extracts of raw red and black beans before and after digestion and reported that there was no significant difference in the ABTS values, and there was only a difference in the extract of black beans when measured by the ORAC method.

It is important to note that, although the total content of PCs detected by HPLC-DAD-FLD was much lower in the case of FF2D, its TPC determined by Folin was somewhat higher than for F2D. In addition, both digests presented higher TPC but lower HPLC-detected PCs than non-digested samples (Table 5 and Table 6). These facts suggested that other substances reactive to Folin were present in the extracts. To analyze this, gel filtration FPLC of the ethanol extracts was performed. After SGID, the presence of molecules with MW < 6.5 kDa strongly increased, and to a much lesser extent, molecules with MW > 10 kDa also increased (Figure 4); the latter presented a much lower abundance than in the case of the fractions soluble in PBS (Figure 2). These analyses demonstrated the presence of other kinds of compounds in the ethanol extracts, such as peptides and amino acids, with higher abundances in FF2D. Therefore, the antioxidant activity of these ethanol extracts showed the contributions of both PCs and peptides and free amino acids that could be solubilized in the extraction conditions.

## 4. Conclusions

The single-step fermentation of yellow pea flour in a liquid medium (14.3% flour dispersion) in a bioreactor (24 h, 37 °C) allowed a product with a pH of 4.4 and a count of about 9 log CFU/g of LABs and significant changes in protein and PCs that could modify the nutritional and bio-functional value of this legume. Partial proteolysis and increased protein digestion after SGID were evident after fermentation, which could be potentially associated with the better nutritional quality of the fermented flour. Also, fermentation produced an increase in extractable PCs, mainly flavonoids, some of which, such as flavan-3-ols, disappeared after SGID, with these digests presenting higher contents of some phenolic acids. The ORAC potency augmentation observed after fermentation could be mainly related to the appearance of small molecules, mostly peptides with sizes lower than 4 kDa. After SGID, fermented flour showed increased ROO· scavenging activity associated with molecules in a broad MW range (>4 kDa to <0.10 kDa).

Subsequent studies will be carried out to study in greater depth the microbial populations responsible for the observed changes, as well as the reproducibility and the effect of seed storage conditions. Also, natural fermentation can improve other biological properties in addition to antioxidant activity, which should be analyzed in order to fully exploit all its benefits. These first results show that natural fermentation could be used as an economical and easy-to-implement tool to achieve a yellow pea ingredient with improved antioxidant properties, whose application in food formulations must be evaluated from a technological point of view.

## Figures and Tables

**Table 1:** Preliminary tests of natural fermentation of yellow pea flour dispersions (36.4% flour *w*/*w*) performed in tubes (10 mL) under different time/temperature conditions.

Fermentation		Proteolysis	Protein Solubility	ORAC
Conditions	Final pH	HD% ^1^	(g SP/100gTP) ^2^	IC_50_ (mg SP/mL)
24 h/30 °C	5.1 ± 0.1 ^b^	16.8 ± 0.7 ^a^	52 ± 5 ^a^	0.071 ± 0.004 ^a^
48 h/30 °C	3.6 ± 0.1 ^a^	17 ± 2 ^a^	54 ± 4 ^a^	0.093 ± 0.004 ^b^
24 h/37 °C	4.7 ± 0.2 ^b^	13 ± 2 ^a^	54 ± 3 ^a^	0.087 ± 0.005 ^b^
48 h/37 °C	3.9 ± 0.1 ^a^	13 ± 2 ^a^	57 ± 8 ^a^	0.066 ± 0.002 ^a^

^1^ Determined in the corresponding dispersions. ^2^ SP: PBS-soluble protein. Different superscript letters (^a,b^) within each column indicate significant differences (Tukey test; *p* < 0.05) among samples.

**Table 2:** Natural fermentation of yellow pea flour dispersions performed in bioreactor: final pH values and microbial counts in YGC, MRS, and NA.

Sample	pH	Microbial Count (log CFU/g)		
		YGC	MRS	NA
F1	6.2 ± 0.1 ^c^	nd	4.2 ± 0.1 ^a^	4.2 ± 0.1 ^a^
FF1	4.75 ± 0.03 ^b^	nd	9.1 ± 0.6 ^b^	8.9 ± 0.6 ^b^
F2	6.29 ± 0.01 ^c^	nd	4.5 ± 0.3 ^a^	4.4 ± 0.2 ^a^
FF2	4.43 ± 0.01 ^a^	nd	9.5 ± 0.3 ^b^	8.8 ± 0.8 ^b^

F1 and F2: pea flour dispersions in conditions 1 and 2, respectively. FF1 and FF2: fermented pea flour dispersions in conditions 1 and 2, respectively. Condition 1: 36.4% *w*/*w* flour, 24 h, 30 °C; condition 2: 14.3% *w*/*w* flour, 24 h, 37 °C. nd: no detected growth in dilution −2. Different superscript letters (^a–c^) within each column indicate significant differences (Tukey test; *p* < 0.05) among samples.

**Table 3:** Composition % of freeze-dried yellow pea flour before and after fermentation in bioreactor.

Sample	Proteins ^1^	Lipids ^1^	Carbohydrates ^1,^*	Fiber ^1^	Ash ^1^	Moisture
F1	17.9 ± 0.3 ^a^	2.3 ± 0.3 ^a^	61.6	15.3 ± 0.8 ^a^	2.8 ± 0.5 ^a^	4.61 ± 0.06 ^ab^
F2	24.2 ± 0.9 ^c^	2.3 ± 0.2 ^a^	55.4	15.2 ± 0.8 ^a^	2.8 ± 0.5 ^a^	4 ± 1 ^a^
FF1	22.1 ± 0.3 ^bc^	2.1 ± 0.1 ^a^	55.4	17.2 ± 0.2 ^ab^	3.1 ± 0.1 ^a^	4.12 ± 0.04 ^a^
FF2	21.9 ± 0.3 ^b^	2.3 ± 0.1 ^a^	53.1	18.8 ± 0.6 ^b^	3.8 ± 0.1 ^a^	5.73 ± 0.08 ^b^

^1^ Contents are expressed as g/100 dry matter. * Carbohydrates were obtained by difference. F1 and F2: pea flour dispersions in conditions 1 and 2, respectively. FF1 and FF2: fermented pea flour dispersions in conditions 1 and 2, respectively. Condition 1: 36.4% *w*/*w* flour, 24 h, 30 °C; condition 2: 14.3% *w*/*w* flour, 24 h, 37 °C. Different superscript letters (^a–c^) within each column indicate significant differences (Tukey test; *p* < 0.05) among samples.

**Table 4:** Protein-related analysis (proteolysis degree ^1^, protein solubility, and antioxidant activity of PBS-soluble fractions ^2^) of yellow pea flour before and after fermentation and after SGID.

Sample	Proteolysis	Soluble Protein	Protein Solubility	ORAC IC_50_	HORAC IC_50_
	HD%	(SP) (mg/mL)	(g SP/100gTP)	(mg SP/mL)	(mg SP/mL)
F1	8.5 ± 0.6 ^a^	2.6 ± 0.1	75 ± 5 ^cd^	0.178 ± 0.019 ^d^	7.4 ± 0.5 ^b^
FF1	17 ± 2 ^b^	2.3 ± 0.1	56 ± 4 ^ab^	0.071 ± 0.007 ^c^	7.7 ± 0.5 ^b^
F1D	45 ± 2 ^c^	4.1 ± 0.6	86 ± 10 ^cd^	0.049 ± 0.003 ^bC^	3.7 ± 0.2 ^a^
FF1D	64 ± 4 ^e^	3.8 ± 0.7	76 ± 10 ^cd^	0.024 ± 0.001 ^aB^	3.8 ± 0.3 ^a^
F2	4 ± 1 ^a^	3.3 ± 0.1	71 ± 2 ^bc^	0.089 ± 0.001 ^c^	7.9 ± 0.9 ^b^
FF2	20 ± 2 ^b^	2.1 ± 0.2	49 ± 3 ^a^	0.033 ± 0.007 ^ab^	7 ± 1 ^b^
F2D	44 ± 3 ^c^	3.5 ± 0.4	88 ± 8 ^d^	0.017 ± 0.001 ^aA^	3.6 ± 0.4 ^a^
FF2D	53 ± 4 ^d^	2.9 ± 0.2	79 ± 5 ^cd^	0.017 ± 0.001 ^aA^	3.6 ± 0.3 ^a^

F1 and F2: pea flour dispersions in conditions 1 and 2, respectively. FF1 and FF2: fermented pea flour dispersions in conditions 1 and 2, respectively. F1D, F2D, FF1D, and FF2D: simulated gastrointestinal digests. Condition 1: 36.4% *w*/*w* flour, 24 h, 30 °C; condition 2: 14.3% *w*/*w* flour, 24 h, 37 °C. ^1^ Determined in the corresponding dispersions. ^2^ Determined in 20 mg/mL dispersions of freeze-dried samples. Different lowercase letters within each column indicate significant differences (Tukey test, *p* < 0.05, all samples). Capital letters (ORAC assay) indicate significant differences (Tukey test, *p* < 0.05; only gastrointestinal digests were included in the analysis).

**Table 5:** Total phenolic content (TPC) and antioxidant activity of 60% ethanol UAE extracts from yellow pea flour (F2), fermented flour (FF2), and the gastrointestinal digests (F2D, FF2D).

Sample	TPC	ORAC	ABTS
	(µg GAE/mL)	IC_50_ (µg GAE/mL)	IC_50_ (µg GAE/mL)
F2	33 ± 1 ^a^	1.2 ± 0.1 ^b^	23 ± 2 ^a^
FF2	96 ± 2 ^b^	1.4 ± 0.3 ^b^	48 ± 10 ^b^
F2D	181 ± 4 ^c^	0.8 ± 0.1 ^a^	29 ± 4 ^a^
FF2D	193 ± 4 ^d^	0.8 ± 0.1 ^a^	22 ± 3 ^a^

Condition 2: 14.3% *w*/*w* F, 24 h, 37 °C. UAE: ultrasound-assisted extraction (15 min, 40% amplitude). GAE: gallic acid equivalent. Different superscript letters (^a–d^) within each column indicate significant differences (Tukey test; *p* < 0.05) among samples.

**Table 6:** Phenolic compound profile of ethanolic UAE extracts from yellow pea flour, fermented flour (FF2), and gastrointestinal digests (F2D, FF2D).

Compound	F	FF2	FD	FF2D
OH-tyrosol	1.7 ± 0.1 ^a^	nd	13.6 ± 0.1 ^c^	7.2 ± 0.1 ^b^
Phenolic acids				
Ellagic acid	0.28 ± 0.02 ^b^	0.44 ± 0.01 ^c^	0.22 ± 0.01 ^a^	0.24 ± 0.01 ^ab^
Gallic acid	0.78 ± 0 ^a^	nd	nd	0.82 ± 0 ^b^
Syringic acid	nd	nd	nd	4.13 ± 0.03
Caffeic acid	2.1 ± 0.5 ^ab^	8.9 ± 0.5 ^c^	0.7 ± 0.2 ^a^	2.9 ± 0.6 ^b^
*p*-Coumaric acid	1.53 ± 0.01 ^d^	0.22 ± 0 ^a^	1.11 ± 0.04 ^c^	0.46 ± 0.01 ^b^
Ferulic acid	0.45 ± 0.09 ^b^	0.19 ± 0.01 ^a^	0.80 ± 0.03 ^c^	0.46 ± 0.02 ^b^
Rosmarinic acid	5.2 ± 0.4 ^b^	6.4 ± 0.1 ^c^	3.30 ± 0.02 ^a^	4.69 ± 0.03 ^b^
Total phenolic acids	10 ± 1	16.1 ± 0.7	6.1 ± 0.2	13.7 ± 0.7
Stilbenes				
Polydatin	26.05 ± 0.04 ^c^	25.61 ± 0.01 ^c^	23.2 ± 0.3 ^b^	22.44 ± 0 ^a^
trans-Resveratrol	2.6 ± 0.1 ^a^	4.8 ± 0.1 ^b^	7.5 ± 0.1 ^d^	6.67 ± 0 ^c^
Total stilbenes	28.6 ± 0.1	30.39 ± 0.09	30.7 ± 0.3	29.11 ± 0
Flavonoids				
Rutin	5.2 ± 0.4 ^a^	13.2 ± 0.7 ^b^	nd	nd
Quercetin-3-glucoside	0.88 ± 0.01 ^a^	1.59 ± 0.01 ^b^	nd	0.96 ± 0.04 ^a^
Kaempferol-3-glucoside	2.3 ± 0.3 ^b^	6.5 ± 0.5 ^c^	0.8 ± 0.1 ^a^	1.1 ± 0.1 ^ab^
Quercetin	nd	3.01 ± 0.01	nd	nd
Procyanidin B1	13 ± 6 ^a^	21 ± 8 ^a^	nd	nd
(+)-Catechin	1.05 ± 0.04 ^a^	1.25 ± 0.04 ^b^	nd	nd
(−)-Epigallocatechin	59.7 ± 0,2	82 ± 6	27 ± 4	0.06 ± 0.08
(−)-Epicatechin	0.55 ± 0.02	nd	27 ± 5 ^c^	nd
(−)-Gallocatechin gallate	nd	nd	6.3 ± 0.2	nd
Naringenin	0.32 ± 0.02	nd	nd	nd
Hesperetin	0.71 ± 0.08 ^a^	1.65 ± 0.07 ^b^	nd	1.71 ± 0.02 ^b^
Total flavonoids	84 ± 6	140 ± 12	40 ± 5	3.86 ± 0.05
Total	125 ± 6	187 ± 12	90 ± 5	47 ± 1

Contents are expressed as µg/g d.m. In the case of FD and FF2D, content refers to the original flour. dm: dry matter. nd: not detected. Condition 2: 14.3% *w*/*w* flour, 24 h, 37 °C. Different superscript letters (^a–d^) indicate significant differences (Tukey test; *p* < 0.05) among samples for each compound.

## Data Availability

The data presented in this study are available on request from the corresponding author. The data are not publicly available due to privacy.
